# Chromosomal mapping of tandem repeats in the Yesso Scallop, *Patinopecten yessoensis* (Jay, 1857), utilizing fluorescence in situ hybridization

**DOI:** 10.3897/CompCytogen.v10i1.7391

**Published:** 2016-03-21

**Authors:** Xuan Li, Zujing Yang, Huan Liao, Zhengrui Zhang, Xiaoting Huang, Zhenmin Bao

**Affiliations:** 1 Key Laboratory of Marine Genetics and Breeding (Ocean University of China), Ministry of Education, Qingdao 266003, China

**Keywords:** Scallop, fosmid, FISH, tandem repeats, chromosome identification

## Abstract

Construction of cytogenetic maps can provide important information for chromosome identification, chromosome evolution and genomic research. However, it hasn’t been conducted in many scallop species yet. In the present study, we attempted to map 12 fosmid clones containing tandem repeats by fluorescence *in situ* hybridization (FISH) in the Yesso scallop *Patinopecten yessoensis* (Jay, 1857). The results showed 6 fosmid clones were successfully mapped and distributed in 6 different pairs of chromosomes. Three clones were respectively assigned to a pair of metacentric chromosomes, a pair of submetacentric chromosomes and a pair of telocentric chromosomes and the remaining 3 clones showed their loci on three different pairs of subtelocentric chromosomes by co-hybridization. In summary, totally 8 pairs of chromosomes of the Yesso scallop were identified by 6 fosmid clones and two rDNA probes. Furthermore, 6 tandem repeats of 5 clones were sequenced and could be developed as chromosome specific markers for the Yesso scallop. The successful localization of fosmid clones will undoubtedly facilitate the integration of linkage groups with cytogenetic map and genomic research for the Yesso scallop.

fluorescence *in situ* hybridization

## Introduction

Chromosome characterization and identification are the very first step to genomic analysis. Construction of cytogenetic maps may enable several types of cytogenetic studies such as chromosomal rearrangements, chromosomal assignment of genes, chromosome identification and others (Wang et al. 2005, Zhang et al. 2007, 2008, Zhao et al. 2015).

The family Pectinidae, with approximately 300 extant species, is widely distributed in world oceans (Waller 2006). Till now, cytogenetic analyses have been performed in only 17 species (Odierna et al. 2006). The chromosome numbers of Pectinidae range from 26 to 38, and the published karyotypes showed that their chromosomes were similar in both size and morphology (Odierna et al. 2006, Leitão and Chaves 2008, Hu et al. 2013). In recent years, chromosome banding techniques have been applied in several scallop species for attempting to achieve chromosome identification. However, most of them failed because the stable chromosome banding patterns were difficult to obtain. NOR-banding (Nucleolus Organizer Region-) has been analyzed in 8 scallop species and only one or two pairs of chromosome can be identified (Insua et al. 1998, 2006, Pauls and Affonso 2000, Gajardo et al. 2002, López-Piñón et al. 2005, Huang et al. 2006, 2007a, Odierna et al. 2006). C-banding and fluorescence banding could illustrate the heterochromatin regions on chromosomes. Nevertheless, having been conducted in 8 kinds of scallops, these two approaches are considered not suitable for chromosome identification as well, mainly due to the huge individual differences (Insua et al. 1998, Pauls and Affonso 2000, Gajardo et al. 2002, López-Piñón et al. 2005, Odierna et al. 2006, Huang et al. 2007b, Huan et al. 2010). RE-banding (Restriction Enzyme-) was only reported in *Adamussium colbecki* (Smith, 1902) (Odierna et al. 2006). Yet, it shared a quite similar outcome to C-banding. As a result, the different banding results showed that those methods could neither offer a high enough number of bands nor provide the uniform outcome of different individuals, needed for chromosome identification.

Fluorescence *in situ* hybridization (FISH) can directly show visual images of hybridization loci, therefore, is a powerful tool to define cytogenetic location (Bartlett 2004). Because of its great advantages in specific sequences mapping, FISH has been widely used for locating repetitive sequences in scallops (Insua et al. 1998, 2006, Wang and Guo 2004, López-Piñón et al. 2005, Odierna et al. 2006, Huang et al. 2007a, 2007b, Zhang et al. 2007). There is no doubt that with repetitive sequences such as rDNA and histone genes successfully being mapped to chromosomes, it has made a progress in distinguishing chromosomes. Whereas such probes are still limited for chromosome identification uses based on the fact only a small number of chromosomes could be recognized.

Large insert clones like bacterial artificial chromosome (BAC), fosmid, P1 and so on have already been tested and proven its practicability for FISH localization (Wang et al. 2005, Zhang et al. 2008, Feng et al. 2014, Zhao et al. 2015). And it has been proven to be a viable approach to map genetic loci in bivalve. For instance, in the Zhikong scallop *Chlamys farreri* (Jones et Preston, 1904), fosmid clones showed the high efficiency of FISH mapping and 8 pairs of chromosomes were successfully distinguished (Zhang et al. 2008). More splendidly, based on microsatellite linkage map, an integrated genetic cytogenetic map of the Zhikong scallop was constructed utilizing BAC clones and 17 pairs of chromosomes were identified which helped genomic assembly of this species (Feng et al. 2014).

The Yesso scallop, *Patinopecten yessoensis* (Jay, 1857), is a cold water bivalve and is naturally distributed along the coastline of northern Japan, the Far East of Russia and the northern Korean Peninsula (Waller and Shumway 1991). It is a species of great economic importance in China and Japan. The production has exceeded 200k tons in 2010 (FAO website; http://www.fao.org/fishery/culturedspecies/Patinopecten_yessoensis). Some genetic researches, such as gene expression analysis, development of SSRs and construction of a linkage map, have been conducted on the Yesso scallop (Liu et al. 2009, Wang et al. 2009, Li et al. 2015). Former cytogenetic studies of the Yesso scallop showed it possessed the haploid number (n=19) and a karyotype formula of 3m+5sm+8st+3t (Zhang et al. 2007). In addition, histone H3 gene loci and rDNA loci were located by FISH and were used to discuss the karyotypic evolution in Pectinidae (Huang et al. 2007b, Zhang et al. 2007). Moreover, vertebrate telomere sequence has been used for FISH localization as well (Huang et al. 2007b). Previous studies surely contributed to the work of chromosome identification of *Patinopecten yessoensis*. Yet, more information provided by specific chromosomal markers is still needed for further cytogenetic study. Recently, a fosmid library including 122, 880 clones of *Patinopecten yessoensis* has been constructed in our lab. This library provides enough probes for us to construct a cytogenetic map for the Yesso scallop.

In the present study, to develop chromosome specific markers for chromosome mapping, we selected 12 fosmid clones containing tandem repeats. These anchored fosmid clones were labeled as FISH probes to hybridize to chromosomes of Yesso scallop. We showed the first time that fosmid clones with long tandem repeats inside can be mapped to *Patinopecten yessoensis* and succeeded in chromosome identification which would be helpful for cytogenetic research in Pectinidae.

## Methods

### Chromosome preparation

Trochophore larvae of *Patinopecten yessoensis* were obtained and handled referring to previous study (Huang et al. 2007b). Chromosome spreads were obtained by dissociating fixed larvae in 50% acetic acid and dropping the cellular suspension onto slides heated to 56°C.

### Selection of fosmid clones and probe labeling

*Patinopecten yessoensis* genome sequencing data (BioProject number PRJNA259405) were subjected to tandem repeat sequences searches using TANDEM REPEATS FINDER (TRF) software (Benson 1999). In addition, the restriction fragments of two-dimensionally pooled fosmid clones were sequenced and generated sequence tags. These sequence tags are assigned to individual fosmid clones according to the method in Oeveren et al. (2011). TRF results were then cross checked with those sequence tags and 12 mono-clones including tandem repeats were selected for probe labeling. Detailed information on tandem repeats is provided in Table 1.

**Table 1.** Primers used for tandem repeats amplification and amplification conditions.

Clone name	Tandem repeats ID	Period size	Copy number	Primer	Primer sequence(5’-3’)	Annealing temperature	Extending time
PF114G13	PY_TR0611036	44	243.9	F-PF114G13 R-PF114G13	GCAAGAACATTTGTCTGCTGA GCGGACTAGGAAAGAGTGATAA	56°C	11min
PF117C11	PY_TR0191169	38	268.5	F-PF117C11 R-PF117C11	ATTAGGCACCGTTGAACAGG GGTATGGCGAGAAGACAGGAT	57.5°C	10min30s
PF9J1	PY_TR0084577	34	269.6	F-PF9J1 R-PF9J1	CATCTAATCACATTCTTACGCACC CTTCACAAGCAGGCAAATCATA	58.5°C	10min
PF105M7	PY_TR0226699	114	81.7	F-PF105M7 R- PF105M7	TGGGATTTGAGTCACGATTT ACAATGGGAACTAGGGATCAT	55°C	10min
PF126O24	PY_TR0180504	20	493.8	F-PF126O24 R- PF126O24	GAACTGAGGCGACATAGACATAG GGAAATAACTTCCCAGAACTGA	56°C	10min
PF115K10	PY_TR0380838	37	289.7	F- PF115K10 R- PF115K10	TCTATTGACAGGGCTACATTTG AACTTGGAAAGAAAGGGGAA	55°C	11min

Plasmid DNA from fosmid clones, with an average insert size of 30-45 kb, was extracted by standard laboratory method (Sambrook and Russell 1989) and labeled with digoxingenin-11-dUTP or biotin-16-dUTP using Dig- or Biotin-Nick Translation Mix (Roche) following the manufacturer’s instruction. Labeled probes were purified by SanPrep PCR products purify kit (Sangon Biotech) and then resolved at a concentration of 5-10 ng/μl in a hybridization solution of 2×SSC, 50% deionized formamide and 10% dextran sulphate.

### FISH and Co-hybridization

FISH experiments were performed following methods previously published (Huang et al. 2007b). DNA of chromosomes was denatured in a mixture containing with 70% formamide and 2×SSC at 76°C for 2 min 30 sec, dehydrated with a series of pre-cool ethanol (70%, 90%, 100%; 5 min each) and air-dried. Hybridization mix was denatured at 90°C for 5 min and cooled rapidly. After incubating with hybridization mix for 16h at 37°C in a moist chamber, slides were washed once in 50% formamide and 2×SSC for 5 min, three times in 2×SSC at 37°C (for 5 min each). Signal detection was performed using anti-digoxigenin-rhodamine (Roche) and fluorescein avidin DOS (Vector). Slides were counterstained with DAPI (4’, 6-diamidino-2-phenylindole) in antifade solution (Vector). Microscopic analysis and capture of chromosome images were carried out using a Leica DM4000B microscope equipped with an epifluorescence system and the appropriate filter sets for fluorescein, rhodamine and DAPI as well as CCD camera. The signals were collected and processed with FISH software (Leica CW4000 CytoFISH Version Y 1.3.1). In each image, in order to show the relative size of chromosomes possessing positive signals, the biggest metacentric chromosome, which could be easily distinguished from the others, was particularly selected as a reference to make comparison with so that the relations between FISH results of different probes in different metaphases can be determined.

Also, co-hybridization was conducted when signals of two different probes were located in the similar chromosomes. The protocol follows the same procedure of regular hybridization. And the hybridization mix with a total volume of 30μl contained 5–10ng/μl of each probe, 50% formamide, 10% dextran sulphate and 2×SSC.

### Tandem repeat sequencing and sequence analysis

Based on tandem repeat sequences scanning from *Patinopecten yessoensis* genome sequencing data, we designed six pairs of primers via PRIMER5 software (Lalitha 2000) to amplify the total length of tandem repeats contained in the 6 fosmid clones that we successfully located by FISH. PCR procedures were conducted following the manufacturer’s instruction of Platinum Taq DNA Polymerase High Fidelity (Invitrogen). Cycling conditions were as follows: 2 min at 94°C (denaturation); 30 cycles of 15s at 94°C, 30s at annealing temperature, and 1min/kb at 68°C for extending. Detailed information about the primers can be found in Table 1. The products were purified with SanPrep PCR products purify kit (Sangon Biotech) for double end-DNA sequencing by ABI3730. Sequences were subjected to sequence similarity searches using BLASTN. All sequences were deposited into Genbank with the accession number listed in Table 2.

**Table 2.** Hybridization and BLASTN results of the mapped 6 clones.

Clone name	Chromosome type*	Location of signals	Accession no.*	Identities
PF114G13	st	Telomeric region of 9q	F: KU041535 R: KU041536	93% 96%
PF117C11	sm	Centromeric region of 6q	F:KU041538	95%
PF9J1	t	Telomeric region of 18q	F: KU041532 R: KU041533	97% 96%
PF105M7	m	Telomeric region 2q	F: KU041534	96%
PF126O24	st	Middle region of 12q	N/A	
PF115K10	st	Centromeric region of 10q	R: KU041537	97%

* m: metacentric, sm: submetacentric, st: subtelocentric, t: telocentric; F: forward sequence, R: reverse sequence

## Results

### FISH signal and distribution

**Figure 1.**
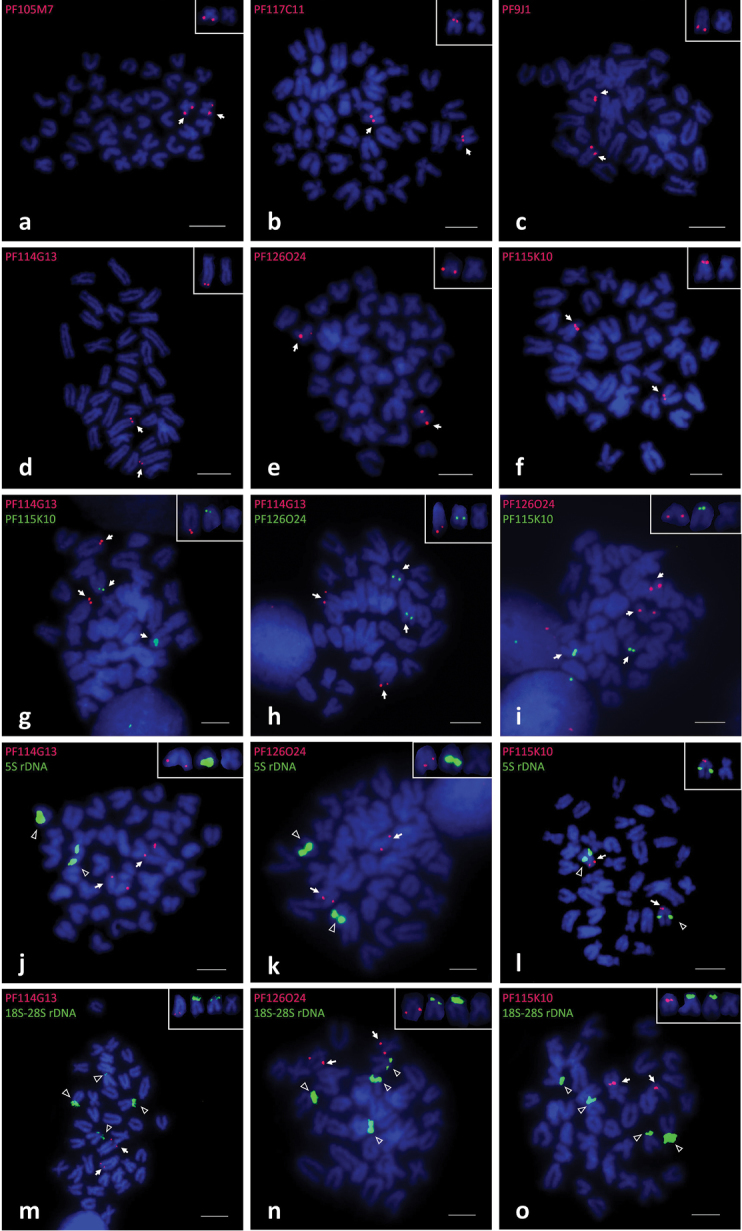
FISH results of fosmid clones on mitotic metaphase chromosomes of *Patinopecten yessoensis*. **a–f**: Mapping of clone PF105M7(**a**), clone PF117C11(**b**), clone PF9J1(**c**), clone PF114G13(**d**), clone PF126O24(**e**) and clone PF115K10(**f**) **g–i** Co-hybridization of clone PF114G13 & PF115K10(**g**), clone PF114G13 & 126O24(**h**) and clone PF126O24 & 115K10(**i**) **j–l** Result of co-hybridization of 3 clones and 5S rDNA sequence, i.e. PF114G13&5S rDNA (**j**), PF126O24&5S rDNA (**k**), PF115K10&5S rDNA (**l**) **m–o** Co-hybridization of 3 clones and 18S-28S rDNA, clone PF114G13 & 18S-28S rDNA (**m**), clone PF126O24 & 18S-28S rDNA(**n**), clone PF115K10 & 18S-28S rDNA (**o**). The insert figure at the top right corner for each of the probes correspond to one chromosomal location showing the labeled chromosomes adjacent to the biggest metacentric chromosome. The arrows indicate positive signals of the clones and the open triangles indicate positive signals of 5S rDNA and 18S-28S rDNA. Scale bars: 10 μm

In this study, 12 fosmid clones were selected for FISH localization and at least 30 metaphases were examined for each probe. Among them were six fosmid clones successfully located on the chromosomes. The remaining 6 clones did not produce any signals, therefore, could not be mapped. Paired and specific signals were observed in the analyzed metaphases and their stability was were proved by repeating FISH procedure more than once. Of the six clones that could be located on the chromosomes with unique loci, clone PF105M7 was hybridized to the telomeric region of the long arm of a pair of metacentric chromosomes (Fig. 1a), clone PF117C11 was the only one with signals mapped to the centromere region of a submetacentric chromosome pair (Fig. 1b), clone PF9J1 was hybridized to the telomeric region of the long arm of a pair of telocentric chromosomes (Fig. 1c).

Three further clones, PF114G13, PF126O24, PF115K10, were mapped to 3 different pairs of subtelocentric chromosomes. Clones PF114G13 (Fig. 1d) was assigned to the telomeric region of the long arms. Clone PF126O24 (Fig. 1e) showed signals on the middle region of the long arms. And as shown in Fig. 1f, clone PF115K10 was mapped to a position quite near the centromere region.

The loci of clone PF105M7, PF117C11 and PF9J1 can be easily distinguished due to the significant differences observed from morphological character of chromosome pairs which they were mapped to. As for the remaining three clones, although locus position diversity was shown, because of similar chromosomal shape and size it was difficult to achieve chromosome separation only according to morphological character. Therefore, co-hybridization of these 3 clones was conducted to confirm their chromosome assignments. As shown in Fig. 1g–h, after co-hybridization, the results confirmed that PF114G13 was located on a different pair of chromosomes with PF115K10 (Fig. 1g) and PF126O24 (Fig. 1h). The co-hybridization result of clone PF126O24 and clone PF115K10 (Fig. 1i) clearly revealed these two clones were mapped to two individual pairs of chromosomes as well.

Further, we co-hybridized 5S rDNA and 18S-28S rDNA with clone PF114G13, PF115K10 and PF126O24 because they were all located on subtelocentric chromosomes. The results of co-hybridization between 5S rDNA and those 3 fosmid clones were displayed in Fig. 1j–l. Clone PF114G13 and PF126O24 showed different chromosome assignment with 5S rDNA (Fig. 1j–k). But clone PF115K10 was mapped on the same pair of chromosomes with 5S rDNA. And Fig. 1m-o demonstrated those 3 fosmid clones were located on the different chromosomes which contained 18S-28S rDNA.

**Figure 2.**
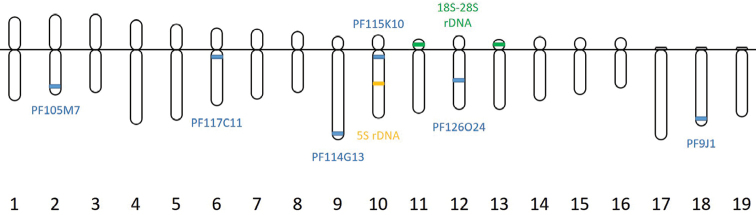
Chromosome ideograms of *Patinopecten yessoensis* showing chromosome assignment of 6 fosmid clones and rDNA. Chromosomes numbering is based on chromosome type and relative length. The blue blocks represent the loci of the 6 clones that have been confirmed by co-hybridization. The orange block represents the loci of 5S rDNA. The green blocks represent the loci of 18S-28S rDNA.

The available data could be used for construction of the karyotypic ideogram of *Patinopecten yessoensis* indicating FISH mapping of the 6 clones and rDNA (Fig. 2). In summary, using these 6 fosmid clones, 6 of 19 chromosomes of Yesso scallop can be identified.

### PCR and tandem repeats sequencing results

The tandem repeats from the 6 mapped fosmid clones were amplified and the sizes of products varying from about 9 to 11kb. The length of those products was identical with TRF results. The PCR products of PF114G13 and PF9J1 were successfully sequenced from both ends. And the products of PF105M7, PF115K10 and PF117C11 were successfully sequenced from the single ends. A BLASTN analysis of the 7 sequences against the *Patinopecten yessoensis* genome sequencing data showed significant sequence matches as we expected and confirmed the existence of tandem repeats (Table 2). The sequencing result of clone PF126O24 was not matched with the tandem repeats sequence but identical to the upstream sequence of the tandem repeats with 96% match percentage, which was caused by the far position between primers and tandem repeats.

## Discussion

Chromosome mapping is an essential step in understanding the genome organization. But together with the small differences in chromosome size and morphology in *Patinopecten yessoensis* and most molluscs, it still remains a challenge for unequivocal identification of each chromosome pairs. Karyotyping and DAPI-banding have been applied to gain more knowledge about chromosomes of *Patinopecten yessoensis* (Komaru and Wada 1985, Huang et al. 2007b), but the results were proven to be less useful for chromosome identification. FISH is a powerful tool which can significantly contribute to this target. However, only histone H3 gene, rDNA and vertebrate telomeric sequence have been mapped to *Patinopecten yessoensis* chromosomes so far (Huang et al. 2007b, Zhang et al. 2007). Vertebrate telomeric sequences were located on the telomeric region of all chromosomes and not suitable for chromosome identification (Huang et al. 2007b). Possessing specific chromosome loci, histone H3 gene and rDNA can be used to identify only one or two pairs of chromosomes (Huang et al. 2007b, Zhang et al. 2007).

Large insert clones like BAC, P1 and fosmid have been already successfully applied in bivalve to reach the goal of chromosome mapping (Wang et al. 2005, Zhang et al. 2008, Feng et al. 2014). In the eastern oyster *Crassostrea virginica* (Gmelin, 1791), 9 of 21 P1 clones, with average size of 75 kb, have been tested and mapped to specific chromosomes (Wang et al. 2005). What is more, fosmid clones which carry a smaller insert size ranging from 30 to 45 kb were used for FISH of *Chlamys farreri* and showed a success rate of 42% (Zhang et al. 2008). In this study, 6 of 12 fosmid clones were successfully assigned to specific chromosomes, indicating a success rate of 50%, which is slightly higher than that in *Crassostrea virginica* or *Chlamys farreri*. Although we shared the close success rate of hybridization, end-sequence information and hybridization results reported in *Chlamys farreri* indicating that fosmid clones containing tandem repeats tended to show multiple signals on chromosomes (Zhang et al. 2008), the multiple signals meant that they were considered not suitable for chromosome mapping. But in our study, we first mapped tandem repeats contained in fosmid clones as unique sequence probes to specific chromosomes in *Patinopecten yessoensis* with positive result that 6 of 12 clones successfully mapped to the chromosomes (50%). Moreover, species-specific *C_0_t*-1 DNA was widely applied during mapping of large-insert clones in order to eliminate nonspecific hybridization in the two researches mentioned above (Wang et al. 2005, Zhang et al. 2008). However, in this study, fosmid clones contained long-size tandem repeats were tested and mapped to specific chromosomes without applying *C_0_t*-1 DNA. As *C_0_t*-1 DNA of *Patinopecten yessoensis* is not necessary during FISH, this indicated that long tandem repeats have great potential to be used for developing unique chromosomes markers in the Yesso scallop.

The tandem repeats sequence we chose for FISH mapping are all mini-satellite DNA which represent about 96% a large portion of tandem repeats in genome of *Patinopecten yessoensis*. Proving its potential for FISH mapping in this study, mini-satellite could be considered as a kind of ideal marker for construction of a cytogenetic map. The tandem repeats from the 6 mapped fosmid clones were amplified and sequenced from the both end. Eight end sequences were generated and the rest did not produce high-quality sequences, therefore, they are not presented here. BLASTN analysis of the 8 sequences against the genome sequence of the Yesso scallop showed significant sequence match with the target sequence which demonstrated the accuracy of whole genome profiling (WGP) method we used for decoding fosmid clones. BLASTN analysis of 7 sequences was conducted against nucleotide collection database on NCBI as well. The results showed that no significant similarity was found for five of them except sequence KU041533 and sequence KU041532 which both came from sequencing results of clone PF9J1. These two sequences were matched to the microsatellite sequence (CFJD036) of *Chlamys farreri* with similarities of 56/63 and 57/63 respectively.

FISH analysis was widely used to establish the relationships between linkage groups and chromosomes in many eukaryotic species such as cucumber and the Zhikong scallop (Ren et al. 2009, Feng et al. 2014). Integrating genetic and cytogenetic maps would be very useful in modifying linkage groups, facilitating whole genome assembly or even detecting chromosome variation in some cases. In the present study, with chromosome assignment of DNA probes of 6 fosmid clones contained tandem repeats as probes, we identified 6 pairs of chromosomes of the Yesso scallop by FISH. In previous study, clusters of 5S rDNA and 18S-28S rDNA were localized on 3 different pairs of subtelocentric chromosomes of the Yesso scallop (Huang et al. 2007b). Therefore, we obtained 18S-28S rDNA and 5S rDNA probes by PCR amplification, labeled with biotin-16-dUTP and applied as a control for the positional relation between fosmid clones and ribosomal DNA. After co-hybridization, 8 of 19 pairs of chromosomes can be distinguished from the others in *Patinopecten yessoensis*. With more fosmid clones successfully localized on chromosomes, it will undoubtedly facilitate construction of cytogenetic maps, assignment of linkage groups and genome assembly for the Yesso scallop.

## Conclusion

In the present study, we identified 6 pairs of chromosomes in the Yesso scallop by FISH using 6 fosmid clones contained tandem repeats as probes. Furthermore, along with mapping of 5S and 18S-28S rDNA, 8 of the 19 chromosome pairs were unequivocally identified. Although the FISH data presented here could not distinguish all chromosomes, these results represent the first step in the development of chromosome specific markers in the Yesso scallop. Ideally, it would be better to have 2 to 4 FISH probes per chromosome arm. Some additional researches are in progress in order to develop more chromosome markers to increase chromosome coverage by localizing repetitive sequences, functional genes and markers from genetic linkage map, etc.

## References

[B1] Bartlett JM (2004) Fluorescence *in situ* hybridization: technical overview. Methods in Molecular Medicine (Springer) 97: 77–87. doi: 10.1385/1-59259-760-2:077 15064486 10.1385/1-59259-760-2:077

[B2] Benson G (1999) Tandem repeats finder: a program to analyze DNA sequences. Nucleic Acids Research 27(2): 573–580. doi: 10.1093/nar/27.2.573 9862982 10.1093/nar/27.2.573 PMC148217

[B3] Feng L Hu L Fu X Liao H Li X Zhan A Zhang L Wang S Huang X Bao Z (2014) An integrated genetic and cytogenetic map for Zhikong scallop, *Chlamys farreri*, based on microsatellite markers. PloS ONE 9: . doi: 10.1371/journal.pone.0092567 10.1371/journal.pone.0092567 PMC3976258 24705086

[B4] Gajardo G Parraguez M Colihueque N (2002) Karyotype analysis and chromosome banding of the Chilean-Peruvian scallop *Argopecten purpuratus* (Lamarck, 1819). Journal of Shellfish Research 21: 585–590. http://d.wanfangdata.com.cn/NSTLQK/NSTL_QKJJ029826074

[B5] Huang X Bao Z Bi K Hu J Zhang C Zhang Q Hu X (2006) Chromosomal localization of the major ribosomal RNA genes in scallop *Chlamys farreri*. Acta Oceanologica Sinica 25: 108–115. http://www.hyxb.org.cn/aosen/ch/reader/view_abstract.aspx?file_no=20060312&flag=1

[B6] Huang X Hu J Hu X Zhang C Zhang L Wang S Lu W Bao Z (2007a) Cytogenetic characterization of the bay scallop, *Argopecten irradians irradians*, by multiple staining techniques and fluorescence *in situ* hybridization. Genes & Genetic Systems 82: 257–263. doi: 10.1266/ggs.82.257 17660696 10.1266/ggs.82.257

[B7] Huang X Hu X Hu J Zhang L Wang S Lu W Bao Z (2007b) Mapping of ribosomal DNA and (TTAGGG)_n_ telomeric sequence by FISH in the bivalve *Patinopecten yessoensis* (Jay, 1857). Journal of Molluscan Studies 73: 393–398. doi: 10.1093/mollus/eym036

[B8] Huan P Zhang X Li F Zhang Y Zhao C Xiang J (2010) Chromosomal localization and molecular marker development of the lipopolysaccharide and beta-1, 3-glucan binding protein gene in the Zhikong scallop *Chlamys farreri* (Jones et Preston) (Pectinoida, Pectinidae). Genetics and Molecular Biology 33: 36–43. doi: 10.1590/S1415-47572010005000015 21637602 10.1590/S1415-47572010005000015 PMC3036087

[B9] Hu L Huang X Zhang L Lu W Bao Z (2013) Progress of Pectinidae Chromosome Study: A Review. Marine Sciences 8: 130–136. http://159.226.158.205/hykx/ch/reader/view_abstract.aspx?file_no=20130822&flag=1 [In Chinese]

[B10] Insua A López-Piñón MJ Méndez J (1998) Characterization of *Aequipecten opercularis* (Bivalvia: Pectinidae) chromosomes by different staining techniques and fluorescent *in situ* hybridization. Genes & Genetic Systems 73: 193–200. doi: 10.1266/ggs.73.193 9880917 10.1266/ggs.73.193

[B11] Insua A López-Piñón MJ Freire R Méndez J (2006) Karyotype and chromosomal location of 18S–28S and 5S ribosomal DNA in the scallops *Pecten maximus* and *Mimachlamys varia* (Bivalvia: Pectinidae). Genetica 126: 291–301. doi: 10.1007/s10709-005-7408-7 16636923 10.1007/s10709-005-7408-7

[B12] Komaru A Wada K (1985) Karyotypes of four species in the Pectinidae (Bivalvia: Pteriomorphia). Venus 44: 249–259.

[B13] Lalitha S (2000) Primer premier 5. Biotech Software & Internet Report: The Computer Software Journal for Scient 1: 270–272. doi: 10.1089/152791600459894

[B14] López-Piñón M Insua A Méndez J (2005) Chromosome analysis and mapping of ribosomal genes by one-and two-color fluorescent *in situ* hybridization in *Hinnites distortus* (Bivalvia: Pectinidae). Journal of Heredity 96: 52–58. doi: 10.1093/jhered/esi001 15598716 10.1093/jhered/esi001

[B15] Leitão A Chaves R (2008) Banding for chromosomal identification in bivalves: a 20-year history. Aquaculture 1 Dynamic Biochemistry, Process Biotechnology and Molecular Biology Volume 2: 44–49. http://www.globalsciencebooks.info/Online/GSBOnline/images/0812/DBPBMB_2(SI1)/DBPBMB_2(SI1)44-49o.pdf

[B16] Liu W Bao X Song W Zhou Z He C Yu X (2009) The construction of a preliminary genetic linkage map in the Japanese scallop *Mizuhopecten yessoensis*. Yi chuan 31(6): 629–637. doi: 10.3724/SP.J.1005.2009.00629 [In Chinese with English abstract] 19586864 10.3724/sp.j.1005.2009.00629

[B17] Li R Zhang R Zhang L Zou J Xing Q Dou H Hu X Zhang L Wang R Bao Z (2015) Characterizations and expression analyses of NF-kB and Rel genes in the Yesso scallop (*Patinopecten yessoensis*) suggest specific response patterns against Gram-negative infection in bivalves. Fish & Shellfish Immunology 44: 611–621. doi: 10.1016/j.fsi.2015.03.036 25842178 10.1016/j.fsi.2015.03.036

[B18] Odierna G Aprea G Barucca M Canapa A Capriglione T Olmo E (2006) Karyology of the Antarctic scallop *Adamussium colbecki*, with some comments on the karyological evolution of pectinids. Genetica 127: 341–349. doi: 10.1007/s10709-005-5366-8 16850238 10.1007/s10709-005-5366-8

[B19] Oeveren J Ruiter M Jesse T van der Poel H Tang J Yalcin F Janssen A Volpin H Stormo KE Bogden R (2011) Sequence-based physical mapping of complex genomes by whole genome profiling. Genome Research 21: 618–625. doi: 10.1101/gr.112094.110 21324881 10.1101/gr.112094.110 PMC3065709

[B20] Pauls E Affonso P (2000) The karyotype of *Nodipecten nodosus* (Bivalvia: Pectinidae). Hydrobiologia 420: 99–102. doi: 10.1023/A:1003989721626

[B21] Ren Y Zhang Z Liu J Staub JE Han Y Cheng Z Li X Lu J Miao H Kang H (2009) An integrated genetic and cytogenetic map of the cucumber genome. PLoS ONE 4: . doi: 10.1371/journal.pone.0005795 10.1371/journal.pone.0005795 PMC2685989 19495411

[B22] Sambrook J Fritsch EF Maniatis T (1989) Molecular Cloning: A Laboratory Manual. Cold Spring Harbor Laboratory Press, Cold Spring Harbor, New York, 2028 pp.

[B23] Waller T Shumway S (1991) Scallops: biology, ecology and aquaculture. Elsevier, 73–73.

[B24] Waller TR (2006) Phylogeny of families in the Pectinoidea (Mollusca: Bivalvia): importance of the fossil record. Zoological Journal of the Linnean Society 148: 313–342. doi: 10.1111/j.1096-3642.2006.00258.x

[B25] Wang Y Guo X (2004) Chromosomal rearrangement in Pectinidae revealed by rRNA loci and implications for bivalve evolution. The Biological Bulletin 207: 247–256. doi: 10.2307/1543213 15616355 10.2307/1543213

[B26] Wang Y Xu Z Pierce JC Guo X (2005) Characterization of eastern oyster (*Crassostrea virginica Gmelin*) chromosomes by fluorescence *in situ* hybridization with bacteriophage P1 clones. Marine Biotechnology 7: 207–214. doi: 10.1007/s10126-004-0051-y 15933900 10.1007/s10126-004-0051-y

[B27] Wang X Song B Qiu X Meng X (2009) Development of EST-SSRs in scallop (*Patinopecten yessoensis*) from sequence database. Conservation Genetics 10: 1129–1131. doi: 10.1007/s10592-008-9726-7

[B28] Zhang L Bao Z Wang S Huang X Hu J (2007) Chromosome rearrangements in Pectinidae (Bivalvia: Pteriomorphia) implied based on chromosomal localization of histone H3 gene in four scallops. Genetica 130: 193–198. doi: 10.1007/s10709-006-9006-8 16909332 10.1007/s10709-006-9006-8

[B29] Zhang L Bao Z Wang S Hu X Hu J (2008) FISH mapping and identification of Zhikong scallop (*Chlamys farreri*) chromosomes. Marine Biotechnology 10: 151–157. doi: 10.1007/s10126-007-9045-x 17955291 10.1007/s10126-007-9045-x

[B30] Zhao B Zhao L Liao H Cheng J Lian S Li X Huang X Bao Z (2015) Mapping toll-like receptor signaling pathway genes of Zhikong scallop (*Chlamys farreri*) with FISH. Journal of Ocean University of China 14: 1075–1081. doi: 10.1007/s11802-015-2643-8

